# Foresight methodologies to unravel the indirect health economic impact of the COVID-19 pandemic on cancer care and management in Belgium studied in the HELICON project: Yasmine Khan

**DOI:** 10.1093/eurpub/ckac129.202

**Published:** 2022-10-25

**Authors:** Y Khan

**Affiliations:** 1 Department of Public Health and Primary Care, Ghent, Belgium; 2 Department of Epidemiology and Public Health, Sciensano, Belgium

## Abstract

**Background:**

The COVID-19 pandemic had indirect effects on segments of the population affected with non-COVID-19 diseases e.g. through delayed care and management of cancer patients. Early diagnosis and appropriate treatment are key to improving patient outcomes and reducing societal and health care costs. Tio support policymakers to anticipate these trends a public health foresight study (PHFS) was done.

**Methods:**

The PHFS follows a structured approach provided through a compact guide and is supervised by PHIRI team members. Its evolution is measured through a template gathering data on the study's contextual information, objective, main target groups, conceptual model, indicators, driving forces, time horizon, spatial unit, identifying uncertainties, scenario logics, scenario type, stakeholders, data, tools and instruments, projection methods, communication strategy/products, and the uptake of results and evaluation.

**Results:**

Several foresight elements were identified. Contextual information on the resources and governance structure were elucidated. The objective was made clear by identifying the topic, general issue, and sub-issues of the initial study. A conceptual model was developed to analyse the interaction of the topic with other aspects that could influence it. The main driving forces, which are factors that influence the studied topic, were then determined through the DESTEP method. Stakeholders were identified and classified through the power-interest matrix.

**Conclusions:**

Establishing a foresight study on the indirect impact of the COVID-19 pandemic on the care and management of cancer patients allows exploring potential and unsuspected issues that may affect society, health care systems, and patients. Those groups should not be considered individually but as an ecosystem continuously interacting, where a decision may affect everyone. This type of information may be of high relevance to policy- and decision-makers in their public health interventions.

